# Novel genotyping approaches to easily detect genomic admixture between the major Afrotropical malaria vector species, *Anopheles coluzzii* and *An. gambiae*

**DOI:** 10.1111/1755-0998.13359

**Published:** 2021-04-01

**Authors:** Beniamino Caputo, Verena Pichler, Giordano Bottà, Carlo De Marco, Christina Hubbart, Eleonora Perugini, Joao Pinto, Kirk A. Rockett, Alistair Miles, Alessandra della Torre

**Affiliations:** ^1^ Dipartimento di Sanità Pubblica e Malattie Infettive Istituto Pasteur Italia‐Fondazione Cenci‐Bolognetti Università di Roma “Sapienza” Rome Italy; ^2^ Wellcome Centre for Human Genetics University of Oxford Headington Oxford UK; ^3^ Global Health & Tropical Medicine Instituto de Higiene e Medicina Tropical Universidade Nova de Lisboa Lisbon Portugal; ^4^ Malaria Programme Wellcome Trust Sanger Institute, Hinxton Cambridge UK; ^5^ MRC Centre for Genomics and Global Health University of Oxford Oxford UK

**Keywords:** ecological speciation, hybridization, malaria vector

## Abstract

The two most efficient and most recently radiated Afrotropical vectors of human malaria – *Anopheles coluzzii* and *An. gambiae* – are identified by single‐locus diagnostic PCR assays based on species‐specific markers in a 4 Mb region on chromosome‐X centromere. Inherently, these diagnostic assays cannot detect interspecific autosomal admixture shown to be extensive at the westernmost and easternmost extremes of the species range. The main aim of this study was to develop novel, easy‐to‐implement tools for genotyping *An. coluzzii* and *An. gambiae*‐specific ancestral informative markers (AIMs) identified from the *Anopheles gambiae* 1000 genomes (Ag1000G) project. First, we took advantage of this large set of data in order to develop a multilocus approach to genotype 26 AIMs on all chromosome arms valid across the species range. Second, we tested the multilocus assay on samples from Guinea Bissau, The Gambia and Senegal, three countries spanning the westernmost hybridization zone, where conventional species diagnostic is problematic due to the putative presence of a novel “hybrid form”. The multilocus assay was able to capture patterns of admixture reflecting those revealed by the whole set of AIMs and provided new original data on interspecific admixture in the region. Third, we developed an easy‐to‐use, cost‐effective PCR approach for genotyping two AIMs on chromosome‐3 among those included in the multilocus approach, opening the possibility for advanced identification of species and of admixed specimens during routine large scale entomological surveys, particularly, but not exclusively, at the extremes of the range, where WGS data highlighted unexpected autosomal admixture.

## 1 INTRODUCTION

Two among the two most efficient and recently radiated Afrotropical vectors of human malaria – *Anopheles coluzzii* and *An. gambiae* (Coetzee et al., 2013) – are morphologically identical but genetically distinct, with most differentiation concentrated in regions of high divergence on the centromeres of the sexual chromosome and of the two autosomes (Ag, 1000G Consortium, 2017). The two species – provisionally named M and S molecular forms (della Torre et al., 2001) – were initially identified by species‐specific SNPs mapping in the IGS multicopy ribosomal region within chromosome‐X centromere (Favia et al., 1997), based on which a few diagnostic PCR and PCR‐RFLP approaches have been developed (Fanello et al., 2002; Santolamazza et al., 2004; Wilkins et al., 2006). A second diagnostic marker, SINE‐X6.1, situated again in the X‐centromeric region and consisting of the exclusive insertion of a transposable element of the SINE‐X200 family in *An. coluzzii* (Santolamazza et al., 2008), is also commonly used for species identification either alternatively or in association with IGS‐markers. Hereafter we will refer to IGS and to SINE for identifications based on IGS‐SNPs and SINE‐X200 insertion, respectively.

*Anopheles coluzzii* and *An. gambiae* are sympatric across west and central Africa although with different biogeographic patterns of habitat segregation (Costantini et al., 2009; Simard et al., 2009), and show strong ecological differentiation, e.g., in larval breeding sites (Cheng et al., 2012; Gimonneau et al., 2012; Kamdem et al., 2012), and in strategies for surviving the dry season (Dao et al., 2014; Huestis et al., 2019). Although viable hybrid progeny are easily generated under laboratory conditions, IGS‐hybrid genotypes are found in nature at a frequency generally <0.2%, thanks to the additive effect of genetic, behavioural and ecological isolating mechanisms (Pombi et al., 2017). The strength of reproductive isolation between *An. coluzzii* and *An. gambiae* varies over space and time leading to periodical breaks in reproductive isolation resulting in extensive hybridization followed by re‐establishment of strong premating barriers (Fontaine et al., 2015; Lee, Marsden, Norris, et al., 2013; Pombi et al., 2017). This incomplete isolation creates opportunities for current gene flow with relevant implication for malaria control, as clearly revealed by extensive evidence of recent adaptive introgression of alleles associated to insecticide resistance between the two species (Clarkson et al., 2014).

Differently from the situation in the rest of the range, at the western edge of the two species range populations showing heterozygous IGS‐patterns are persistently found at frequencies >20% in Guinea Bissau and Senegal (Ndiath et al., 2014; Niang et al., 2014; Nwakanma et al., 2013; Oliveira et al., 2008; Vicente et al., 2017) and up to 7% in The Gambia (Caputo et al., 2008, 2014; Nwakanma et al., 2013). Moreover, in this area, IGS and SINE diagnostics are not always in agreement, as it is believed the case in the rest of the range, suggesting recombination within chromosome‐X centromeric region, which was confirmed by multilocus analysis (Caputo et al., 2016). Indeed, genomic studies have revealed that in coastal Guinea Bissau extensive asymmetric introgressive hybridization from *An. coluzzii* to *An. gambiae* has eroded the major genomic islands of divergence on chromosomes‐2 and −3, but to a far lesser extent on chromosome‐X (Caputo et al., 2016; Lee, Marsden, Norris, et al., 2013; Marsden et al., 2011). Impacts of massive introgressive hybridisation may range from genomic erosion and species collapse to rapid adaptation and speciation (Seehausen et al., 2008; Baack & Rieseberg, 2007; Nolte & Tautz, 2010). Microsatellite and WGS analyses suggested that the coastal region of Guinea‐Bissau is colonized by a stable “hybrid form” – characterised by an *A. gambiae*‐like sex chromosome and by massive introgression of *An. coluzzii* autosomal alleles. This coastal “hybrid form” appears to be separated from genetically distinct *An. gambiae* inland populations by an intermediate area dominated by *An. coluzzii* (Vicente et al., 2017). The consequences of this partitioning on malaria transmission have still to be investigated, but preliminary evidence showed that the coastal “hybrid form” in Guinea Bissau has a lower anthropophylic tendency compared to inland populations (Vicente et al., 2017) and may be resilient to insecticide resistance traits. Intriguingly, a similar pattern of genetic partitioning was reported also between coastal and inland *An. gambiae* populations along the Gambia River (Caputo et al., 2014).

Results from the *Anopheles gambiae* 1000 Genomes (Ag1000G) project showed that a population from coastal Guinea‐Bissau carried a mixture of *An. coluzzii* and *An. gambiae* alleles on all chromosomes and grouped all individuals together in a single population separated from other West African populations of either species (Ag, 1000G Consortium, 2017). Unexpectedly, additional results from the same project revealed that a population in coastal Kenya, at the eastern edge of *An. gambiae* range also carried a mixture of *coluzzii*/*gambiae* alleles on all chromosome arms (except for the 4 Mbp region of chromosome‐X centromere) despite the *An. coluzzii* range not extending east of the Rift Valley. Further analyses and population sampling are required to assess whether either historical admixture with *An. coluzzii* populations, and/or introgression with other species, and/or retention of ancestral variation are responsible for this unexpected pattern.

The above data clearly demonstrate that a simple *gambiae*/*coluzzii* species dichotomy based on the widely used X‐centromeric diagnostic approaches is not sufficient to capture the rich diversity and complex histories of contemporary populations. There is a need for novel, easy‐to‐implement and inexpensive molecular tools with higher ability to detect possible introgression, to be applied on large samples collected within epidemiological or applied field surveys. To this aim, Lee et al. (2013) developed a Divergence Island SNP (DIS) mass‐spectrometry genotyping assay based on 15 SNPs mapping on the centromeric regions of chromosome X (*N* = 7), chromosomal arms‐2L (*N* = 5) and –3L (*N* = 3) and reported to be fixed in the two species (Stump et al., 2005; Turner et al., 2005; White et al., 2010). “Pure” species, F1 and backcross hybrids were arbitrarily identified allowing two mismatched calls to provide reliable estimates of hybridization. This approach allowed the authors to study introgression between *An. coluzzii* and *An. gambiae* even in the absence of huge sequencing capacities and provided important evidence of higher than expected gene flow between the two species as well as of fluctuating hybridization in both time and space (Lee, Marsden, Norris, et al., 2013; Sanford et al., 2014; Norris et al., 2015; Hanemaaijer et al., 2018). While DIS represented a great advance compared to conventional diagnostic assays, allowing the better detection of F1 and backcross hybrids without the need of genome sequencing, it has a few pitfalls, mainly due to the fact that SNPs were chosen based on samples from a limited geographical range, before data from AG1000G project became available. First, none of the 15 SNPs map on chromosomal arm 2R and 3R. Second, the SNPs were ascertained based on a restricted geographical range in west Africa and Cameroon, overlooking the species‐specific diversity in central and east Africa. Third, all SNPs map in relatively small centromeric regions, with presumably high linkage within each chromosomal arm.

The main aim of this study was to develop novel, easy‐to‐implement tools for genotyping *An. coluzzii* and *An. gambiae*‐specific ancestral informative markers (AIMs) identified by the Ag1000G project (Ag, 1000G Consortium 2017). First, we took advantage of the large set of Ag1000G data covering most of the distribution range of the two species, in order to develop a multilocus approach to genotype a number of unlinked AIMs on all chromosome arms valid across the species range. Second, we tested the multilocus assay on samples from Guinea Bissau, The Gambia and Senegal, three countries spanning the high hybridization zone, where single‐locus species diagnostic assays are problematic due to the putative presence of a novel “hybrid form”. Third, we developed an easy‐to‐use cost‐effective end‐point PCR approach for genotyping two AIMs among those on chromosome‐3 included in the multilocus approach, opening the possibility for advanced identification of species and of admixed specimens in routine large scale entomological surveys.

## 2 MATERIALS AND METHODS

### 2.1 Computational ascertainment of diagnostic variants

In order to take into account the maximum possible genetic diversity within *An. coluzzii* and *An. gambiae* genetic pools, diagnostic variants were ascertained based on genomic data from Ag1000G project Phase‐1 data set (Ag, 1000G Consortium, 2017) and from Neafsey et al. (2010), which included sympatric populations from Mali). The latter populations were added in order to identify markers on chromosome arm 2L, as *An. coluzzii* individuals from the Ag1000G Phase 1 data set experienced introgression in the pericentromeric region of this chromosome driven by the *kdr* mutation. Overall, allele frequency difference (DAF) was calculated from 675 individuals from six countries (genotyped in Phase 1 of Ag1000G, i.e., *An. coluzzii* from Burkina‐Faso and Angola and *An. gambiae* from Angola, Burkina‐Faso, Guinea, Gabon, Cameroon, Uganda) spanning a broad geographic range from western to eastern Africa (Ag, 1000G Consortium, 2017). Different DAF cutoffs were calculated in order to be able to find at least 30 diagnostic variants for each chromosomal arm. In the case of SNPs on chromosomal arm 2L, selection was initially carried out based on the three nonintrogressed *An. coluzzii* individuals from Ag1000G and all *An. gambiae* populations, and later selecting only variants matching with the variants with DAF ≥ 0.9 in the Malian populations genotyped in Neafsey et al., (2010). Match with variants with DAF ≥ 0.9 in the Malian populations was also carried out for chromosome‐X variants in order to take into account this additional pool. This was not possible for variants on chromosome arms 2R, 3R and 3L as no matches were present between variants found in WGS data from Ag1000G and the chip array by Neafsey et al., (2010).

### 2.2 Mass spectrometry assay design and validation

To allow optimal assay design, all SNPs selected from the above analysis were inspected for polymorphisms in 250 bases of the 5′ and 3′ flanking sequences with respect to the Ag1000G Phase‐1 data. We only included those flanking polymorphisms where three or more chromosomes were identified carrying the minor allele (MAF > 0.2%). Target SNPs and flanking sequences with polymorphisms identified using standard IUPAC single‐letter notation were formatted for the Agena (formerly Sequenom) MassARRAY Assay Design v3.1 Software (Agena Bioscience).

The successfully designed assays were tested on the Agena iPLEX platform on 343 individuals from nine Ag1000G populations and on 26 individuals from G3 laboratory colony (Table S1). Subsequently, a selection of a final set of assays was undertaken using the following criteria: (a) Performance in preliminary multiplex assays, (b) maximization of distribution on different chromosome arms, (c) location within coding regions, (d) maximization of physical distance between variants, and (e) maximization of the number of assays that fit into a single multiplex. This resulted in a set of 35 SNPs (Table S2 and Supplementary Material S1) for validation on the Agena iPLEX platform using the above mentioned mosquito samples included in Ag1000G.

The subset of validated SNPs was used to genotype a total of 564 indoor‐resting *An. coluzzii* and *An. gambiae* individuals collected in the HHZ along a west to east transect from coastal to inland The Gambia and Senegal (*N* = 188; [Caputo et al., 2008, 2011]) and from coastal to inland Guinea‐Bissau (*N* = 376; [Vicente et al., 2017]), identified based on chromosome‐X linked IGS‐diagnostic marker (Fanello et al., 2002) and, in case of specimens from Guinea Bissau, also by the species‐specific insertion of a SINE‐X200 in the same centromeric region of chromosome‐X (Santolamazza et al., 2008).

Eventually, to further validate species‐specific variants included in the final assay, allele frequency difference was calculated in silico on Ag1000G Phase2 data. This allowed the evaluation of DAF on a total of 938 specimens from eight *An. gambiae* and five *An. coluzzii* populations. Allele frequencies were calculated by python module scikit‐allel v.1.2.1 (https://zenodo.org/badge/latestdoi/7890/cggh/scikit‐allel).

### 2.3 Agena iPLEX mass‐spectrometry assay

Genotyping of samples was undertaken using the Agena iPLEX platform as described in Rockett et al. (Malaria Genomic Epidemiology Network, 2014) and Fabrigar et al., (2015) using primer‐extension preamplified gDNA samples. For specimens from The Gambia whole genome amplification was performed directly on mosquito fragments, such as single legs or wings. Initial genotype calls were assessed using cluster plots viewed on the Agena MassARRAY TYPER v4.0.22 software. Further QC of the data included comparison with Ag1000G data, pass rates of assays across the sample sets, pass rates of samples the set of “valid” assays.

### 2.4 Development of a PCR approach

**FIGURE 1:**
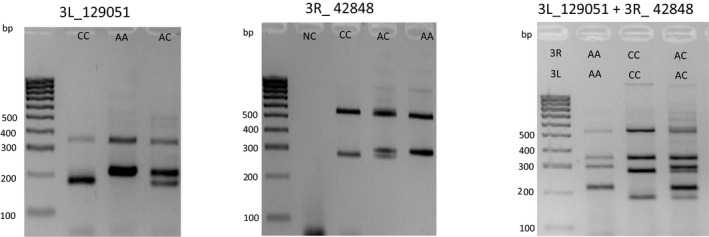
Banding patterns obtained for 3L_129051 (left) and 3R_42848 (centre) loci, genotyped separately and together (right) on a 2.5% agarose gel. In first left lane HyperLadder 100 (Bioline, UK). A, *An. coluzzii*‐specific; Allele C, *An. gambiae*‐specific

Based on results obtained from the mass‐spectrometry assay, sequences flanking the 7 SNPs on chromosome‐3 were scored in order to identify those most suitable ones to design primers for the development of an easy‐to‐use and cost‐effective end‐point PCR‐ approach to easily detect signatures of autosomal introgression. SNPs from chromosome arms 2L and 2R were excluded from the design since they are heavily influenced by the introgression of the *kdr* mutation from *An. gambiae* to *An. coluzzii* (Ag, 1000G Consortium, 2017) and the wide presence of chromosomal inversions (Pombi et al., 2008), respectively. Primers for a Tetra‐ARMS‐PCR for loci 3L_129051 and 3R_42848 on chromosome arms 3L and 3R, respectively, were designed using the online program PRIMER1 (http://primer1.soton.ac.uk/primer1.html) developed by (Ye et al., 2001) and specificity of primers was checked using BLAST (http://www.ncbi.nlm.nih.gov/blast). Primer sequences, fragment length and annealing temperature for amplification of the two loci are provided in Table 1. PCRs for the two loci were optimized separately in a 25 µl PCR reaction performed on a BIORAD C1000. Results were visualized on a 2.5% agarose gel stained with Midori Green Advance (NIPPON Genetics Europe) as shown in Figure 1.

**TABLE 1:** PCR amplification of 3L_129051 and 3R _42848 loci. Primer sequences, annealing temperature (Ta, °C), length of universal outer and allele‐specific inner PCR‐ products (bp)

Locus	Ta (°C)	Primer	Sequences (5'−3')	Product length
3L_129051	64	3L_OUT_fw	AAACACATTTGTGCTTCATTAGTTCCGTG	349
		3L_OUT_rev	TTTCCAAAATATCAACGAAAACCCCTAG	
		3L_IN_fw (allele C)	CGACTTCACATCAAAATTGACCATCAcTC	184
		3L_IN_rev (allele A)	ATACGGAAAAAGGCACATTTTCGgTT	219
3R_42848	64	3R_OUT_fw	TAATGGTTTTTAAGCTTTCCTTTGCCTC	539
		3R_OUT_rev	GAAAACAGATCGATACTCACAAACCGTT	
		3R_IN_fw (allele A)	GATTTAACGCGATGCAATTTCCAcTA	311
		3R_IN_rev (allele C)	GAACCATTTTCCCGTCATTATCAgTG	280

Note

Lower case nucleotides in inner primer sequences indicate intentionally introduced mismatches to enhance specificity. Allele A, *An. coluzzii*‐specific; Allele C, *An. gambiae*‐specific.

PCR reactions were performed on 2 μl of template DNA extracted from a single mosquito (approximately 2–5 ng of DNA). For locus 3L_129051 PCR was performed using 1 unit of Bioline Taq polymerase, 10× PCR Buffer Bioline, 0.08 mM of each dNTP, 2.5 mM MgCl_2_, 0.16 µM of each outer primer, 0.65 µM of inner reverse primer and 0.9 µM of inner forward primer. PCR‐reaction for locus 3R_42848 was set up using 2 units of Bioline Taq polymerase, 10× PCR Buffer Bioline, 0.08 mM of each dNTP, 4 mM MgCl2, 0.2 µM of each outer primer, 0.8 µM of inner reverse primer and 0.6 µM of inner forward primer. For the two PCR assays, thermocycler conditions were 94°C for 10 min followed by 35 cycles of 94°C for 30 s, 64°C for 30 s and 72°C for 1 min, with a final elongation at 72°C for 10 min. When high quality DNA was available it was possible to perform the two assays in a single reaction using 10x PCR Buffer Bioline, 0.08 mM of each dNTP, 2.5 mM MgCl2 and 2 units of Taq polymerase in a 25 µl reaction. Primer concentrations were maintained the same as in single‐locus reactions while quantity of DNA was increased to 3 µl.

Both PCR‐assays were tested on *An. coluzzii* and *An. gambiae* samples from coastal and inland Guinea Bissau for which it was possible to compare PCR and MassArray genotyping results, as well as on specimens from Burkina Faso (Koubri) for which no previous MassArray genotyping data were available. All these specimens were also genotyped for both X‐centromeric diagnostic markers (IGS and SINE) used to identified samples from Guinea Bissau, The Gambia and Senegal (see above). For a subset of individuals, including all the specimens showing unclear or discordant results between PCR and mass spectrometry assay, PCR products were purified using the SureClean protocol (Bioline) and sequenced at Eurofins Genomics GmbH.

## 3 RESULTS

### 3.1 Development and validation of Agena iPLEX mass‐spectrometry assay

A total of 337 species‐specific variants were identified based on different DAF cutoffs for each chromosomal arm (i.e., 1 for chromosome‐X; 0.986 for chromosome arms 2R – 2L – 3R; 0.978 for chromosomal arm‐3L; Supplementary Material S1) in order to obtain at least 30 variants/arm (Supplementary Material S1). Of these 337 variants, six matched with the diagnostic SNPs (*N* = 15) used in the DIS method (Lee, Marsden, Nieman, et al., 2013), two did not pass all the quality filters in the WGS Ag1000G data and the remaining 7 had DAF values below the cut‐off applied in the present study.

Primers were successfully designed for 122 species‐specific variants and split into four multiplexes (Supplementary Material S1). The remaining 216 SNPs failed assay design primarily due either to failure in satisfying the assay constraints or to polymorphisms in possible primer positions (Supplementary Material S1). Mass spectrometry genotyping was successful for 104 species‐specific variants (Supplementary Material S1) and of these, 35 were arranged in a single multiplex mass‐spectrometry assay (Table S2). Out of the latter 35 diagnostic variants, 29 (including three variants also present in the DIS method; (Lee, Marsden, Nieman, et al., 2013; Supplementary Material S1) were successfully genotyped in >75% of tested individuals. Among these 29 variants, 10 mapped on chromosome‐X centromere, seven on chromosome‐2L centromere, three on chromosome‐2R, four on chromosome‐3L centromere and five on chromosome‐3R telomere.

Out of the 29 selected variants, two (3L_367248 and 3R_50590) showed a < 99% concordance between results from Illumina genotyping and from mass‐spectrometry assay (*N* = 229 individuals) (Table S3) and were thus considered not reliable and excluded from the final mass spectrometry assay. It is interesting to note that six out of 7 2L‐variants – which, unlike those on other chromosome arms were selected based on data from a Malian *An. coluzzii* population only (Neafsey et al., 2010) – were virtually fixed (>99% frequency) in *An. gambiae* populations from BF, CM, GA, GN, UG (Table S3). Frequencies of *An. gambiae* specific alleles of these six loci were >85% also in *An. coluzzii* AO and BF populations due to introgression driven by selection on the insecticide‐resistance *kdr* mutations (Ag, 1000G Consortium, 2017). The seventh 2L‐locus (2L_1947574) was highly polymorphic (24% *A. coluzzii* allele) in *An. gambiae* from UG and was conservatively excluded from the final mass‐spectrometry assay.

Of the 26 most reliable species‐specific variants included in the final mass‐spectrometry assay, 10 mapped in a 5.5 Mb region on chromosome‐X centromere, six in a 2.2 Mb region on chromosome‐2L centromere, three in a 9.5 Mb region on chromosome‐2R, three in a 251 Kb region on chromosome‐3L centromere and four in a 37 Kb region on chromosome‐3R telomere (Table 2).

**TABLE 2:** *Anopheles coluzzii* and *An. gambiae* species‐specific variants included in mass‐spectrometry assay. Additional sequence and primer design data provided in Supporting Information Material

Chromosome	Chromosome	GENE ID	*An. coluzzi*	*An. gambiae*
Arm	Position	(AGAP)	Allele	Allele
X	18758300	AGAP000974	C	T
X	19631625	AGAP001022	C	G
X	20014696	AGAP001039	A	C
X	20128328	AGAP001043	C	T
X	20129288	AGAP001043	G	T
X	22164043	AGAP001061	C	T
X	22798648	AGAP001073	A	C
X	23468268	AGAP001082	A	T
X	24229846	AGAP001094	A	G
X	24266728	Intergenic	A	T
2L	209536^a^	AGAP004679	G	A
2L	927247	Intergenic	A	C
2L	1274353^a^	AGAP004691	A	G
2L	1418210	AGAP004692	C	T
2L	1776348	Intergenic	C	G
2L	1947574	AGAP004696	A	G
2L	2431005^a^	AGAP004707	C	T
2R	8368731	AGAP001684	A	C
2R	17854287	AGAP002232	C	A
2R	49438586	AGAP013121	C	T
3L	129051	AGAP010311	C	C
3L	193173	AGAP010312	G	A
3L	367248	AGAP010314	A	C
3L	380974	AGAP010314	A	C
3R	34264	AGAP007732	A	G
3R	38161	AGAP007733	A	G
3R	42848	AGAP007732	A	C
3R	50590	Intergenic	T	G
3R	71973	Intergenic	C	A

Grey shading indicates SNPs excluded from the final assay.

^a^ SNPs included also in the DIS assay proposed by Lee, Marsden, Norris, et al. (2013).

Computation of allele frequency difference on *An. gambiae* (*N* = 655) and *An. coluzzii* (*N* = 283) specimens, including Phase‐1 and Phase‐2 Ag1000G specimens, produced allele frequencies of the 26 SNPs similar to those calculated from Phase‐1 data set and used as DAF cutoffs for each chromosomal arm (Table S4).

We arbitrarily decided to establish a minimum number of consensus between species‐specific variants to define pure *An. gambiae* (Ga) and *An. coluzzii* (Co) multilocus genotypes, as well as F1 (Co/Ga) and admixed (adm) ones, based on the mass‐spectrometry assay. In agreement with the different DAF cutoffs used for the selection of included loci, no discordant SNP was allowed for loci on chromosome‐X (DAF cutoff = 1), while a flexibility of 1 discordant species‐specific variant was allowed for the 10 autosomal loci on chromosome arms 2R, 3R and 3L, in order to account for low levels of intraspecific autosomal polymorphism (DAF cutoff > 0.978). Loci on chromosomal arm 2L were not considered to define species due to the widespread introgression from *An. gambiae* into *An. coluzzii* of the 2L centromeric region where the *vgsc* gene carrying possibly a *kdr* mutation is located (Ag, 1000G Consortium, 2017). Thus specimens were classified as pure Co and Ga when 10/10 loci on chromosome‐X and 9/10 loci on autosomes were genotyped as *An. coluzzii* or *An. gambiae*, respectively; as F1 when 10/10 loci on chromosome‐X and 9/10 loci on autosomes were at the heterozygous state and as adm when discordances among loci were present. Analysis of the mass‐spectrometry genotyping of the 336 (out of 343) successfully genotyped individuals from the nine Ag1000G populations (Table S1) showed some level of admixture in all populations, with the exception of AO and CM (Figure S1). This ranged from ≤5% in BF, GN and UG, to intermediate levels in GM (17%) and GA (29%), to 100% in GW and KE. No individuals with all 26 species‐specific variants at the heterozygous state were found, except that in the G3 laboratory colony, in which level of admixture was >80%. Overall this novel multilocus approach allowed the authors to highlight features fully consistent with Miles et al. (Ag, 1000G Consortium, 2017; Figure S1): (a) admixture on chromosome‐X in populations from GW (53%), GN (4%), UG (1%) and in the laboratory colony (54%), (b) introgression of the centromeric region of chromosome arm 2L in *An. coluzzii* from AO and BF, and (c) a complex pattern of admixture in GW and KE and in the laboratory colony.

### 3.2 Genotyping of *An. coluzzii* and *An. gambiae* field populations

The 26 species‐specific variants included in the Agena iPLEX mass‐spectrometry assay were genotyped in 564 specimens from the westernmost extreme of *An. coluzzii* and *An. gambiae* range, where introgression between the two species is known to be widely present. Considering only specimens for which amplification failed in ≤1 locus/chromosome, 95%, 55% and 83% of specimens from Guinea Bissau (*N* = 358/376), The Gambia (*N* = 77/140) and Senegal (*N* = 40/48) were successfully genotyped. The lower success rate for specimens from The Gambia and Senegal can be explained by the fact that genotyping was performed directly on body parts (legs, wings) preserved >10 years in silica gel, and not on extracted DNA as in the case of samples from Guinea Bissau. Despite this, >50% of all the species‐specific variants, as well as >50% of loci/chromosome, were successfully amplified in 86% of specimens from The Gambia, highlighting the potentiality of the approach to be applied to suboptimal DNA templates (i.e., old samples and/or without DNA extraction).

**FIGURE 2:**
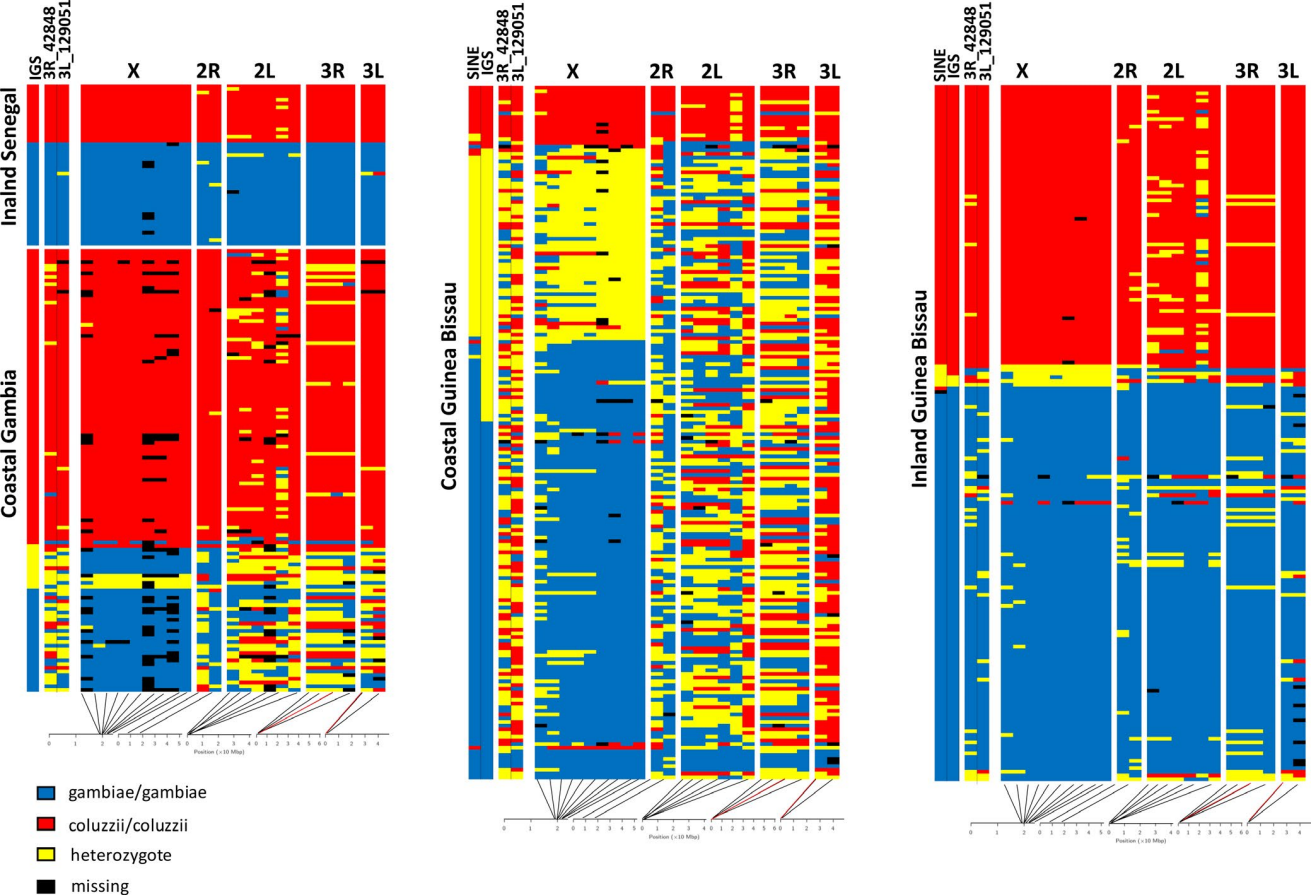
Results of *Anopheles coluzzii* and *An. gambiae* bi‐ and multilocus genotyping at the western extreme of the species range. Only specimens for which >50% of species‐specific variants per chromosome were successfully genotyped are included. Rows represent individual mosquitoes (grouped by geographical origin. Columns represent ancestral informative markers (AIMs; red, *An. coluzzii*; blu, *An. gambiae*; yellow, heterozygote as in Ag, 1000G Consortium, 2017) genotyped by novel multilocus assay grouped by chromosome arm. Far left columns show: columns 1–2 = species assignment according to species diagnostic PCR (IGS – Fanello et al. (2002) – for samples from Senegal and The Gambia; IGS and SINE – Santolamazza et al. (2008) – for samples from Guinea Bissau); 3–4 = species assignment according to 3R_42848 and 3L_129051 AIM loci genotyped by the novel PCR assays. Lines at the lower edge show the approximate physical locations of the AIM SNPs on each chromosomal arm with SNPs 3R_42848 and 3L_129051 indicated in red

Table 3 and Figure 2 show the results of multilocus mass‐spectrometry assay with reference to 23/26 SNPs (three species‐specific variants, X‐22164043, 2R‐49438586 and 3L‐380974, with pass‐rate <75% in samples from Gambia/Senegal are excluded from the analyses) for 533 specimens from Guinea Bissau, Gambia and Senegal with >50% of species‐specific variants overall and per chromosome successfully amplified (Table S5). Samples are classified as pure *An. coluzzii* (Co) or *An. gambiae* (Ga), Co/Ga hybrid (F1) and admixed applying the same criterion described as described above with two differences: (a) The six loci on chromosomal arm 2L are not excluded, since no signs of introgression were detected for the *vgsc* gene (which could have indicated linkage with *kdr* alleles) – with the exception of AIM 2L_1776348 which is highly introgressed in *An. coluzzii* but unlikely due to linkage with *kdr* alleles, as an adjacent AIM (2L_2431005) shows a completely different pattern; (b) a flexibility of two instead of one discordant species‐specific variants is allowed for autosomal loci (as in Lee, Marsden, Nieman, et al., 2013; Lee, Marsden, Norris, et al., 2013) to account for the larger number of loci considered due to inclusion of the 6 chromosome‐2L loci. Thus, specimens were classified as pure Co and Ga when 9/9 loci on chromosome‐X and 12/14 loci on autosomes were genotyped as *An. coluzzii* or *An. gambiae*, respectively; as F1 when 9/9 loci on chromosome‐X and 12/14 loci on autosomes were at the heterozygous state and adm when more discordances among loci were present.

**TABLE 3:** Comparison between species ID obtained using the X‐diagnostic marker (IGS) or the multilocus mass‐spectrometry assay. Only specimens for which >50% of species‐specific variants per chromosome were successfully genotyped are included. Specimens are classified as follows: (i) based on the nine loci on chromosome‐X: Co and Ga when all 9/9 loci correspond to *An. coluzzii* or *An. gambiae*, respectively, F1 when all loci are at the heterozygous state and adm when discordances among loci are present; (ii) based on 9 loci on chromosome X + the 14 loci on autosomes: Co and Ga when ≥12/14 autosomal loci correspond to *An. coluzzii* and *An. gambiae*, respectively, F1 when ≥12/14 autosomal loci are consistently found at the heterozygous state and adm when discordances among loci are present

Country	IGS	X (9 loci)				Con‐cordance	X(9 loci) + autosomes (12 out of 14 loci)				Con‐cordance	Total
		Co	Ga	F1	Adm		Co	Ga	F1	Adm		
Coastal Guinea Bissau	Co	14	–	–	3	71%	8	–	–	9	46%	17
	Ga	–	53	–	36		–	–	–	89		89
	Co/Ga	–	14	18	42		–	–	1	73		74
	Total	14	67	18	81		8	–	1	171		180
Inland Guinea Bissau	Co	76	–	2	1	92%	59	–	–	20	75%	79
	Ga	–	95	–	12		–	80	–	27		107
	Co/Ga	–	–	1	2		–	–	–	3		3
	Total	76	95	3	15		59	80	–	50		189
Coastal Gambia	Co	78	–	–	2	90%	59	–	–	21	59%	80
	Ga	–	26	–	2		–	1	–	27		28
	Co/Ga	1	7	3	1		1	–	–	11		12
	Total	79	33	3	5		60	1	–	59		120
Inland Senegal	Co	16	–	–	–	100%	15	–	–	1	95%	16
	Ga	–	28	–	–		–	27	–	1		28
	Co/Ga	–	–	–	–		–	–	–	–		–
	Total	16	28	–	–		15	27	–	2		44

Results show high levels of concordance between IGS‐based identification and results of the genotyping of the nine loci on chromosome‐X in all populations (92%, 90% and 100% in inland Guinea Bissau, The Gambia and Senegal, respectively), except that from coastal Guinea Bissau, where concordance drops to 71%, largely due to IGS‐ *An. gambiae* specimens with admixed multilocus genotypes (Table 3). Interestingly, a pure multilocus Ga genotype characterises 7/12 and 14/74 IGS‐hybrids from the Gambia and coastal Guinea Bissau, respectively.

When results from genotyping of the 23 loci are taken into account, concordance remains high in the inland Senegal population (95%), but is reduced to 46%, 75% and 59% in populations from coastal, inland Guinea Bissau and coastal Gambia, respectively. In coastal populations from Guinea Bissau and The Gambia the proportion of individuals showing admixed multilocus genotypes with pure IGS‐genotype is higher in Ga (100% and 96%, respectively) than in Co (53% and 26%).

### 3.3 Development of a PCR approach for chromosome‐3 SNP genotyping

The novel PCR approach developed to genotype two species‐specific autosomal SNPs (3L_129051 and 3R_42848) was validated on 106 specimens from across Guinea Bissau, previously analysed by the novel mass‐spectrometry assay, and tested on 146 *An. coluzzii* and 39 *An. gambiae* specimens from Burkina Faso, where no/low admixture is expected (Pombi et al., 2017). For all Guinean specimens, DNA quality was sufficient to allow genotyping of both loci in a single PCR reaction, while for 50% of the specimens from Burkina Faso each locus had to be amplified separately, due to low DNA quality affecting efficacy of simultaneous amplification of the two loci.

In Guinea Bissau samples, results of PCR‐genotyping of both loci are highly concordant (>97%) with results obtained by mass‐spectrometry assay (Table S6). Three (2.8%) discordances are observed for locus 3L‐129051: sequences of the PCR‐products allowed to identify one genotyping error in the mass‐spectrometry assay and two in the PCR‐genotyping approach, one of which was explained by a polymorphism next to the species‐specific SNP genotyped (Table S7). For the single mismatch observed for locus 3R‐42848, sequencing confirmed the mass‐spectrometry assay results.

Table 4 shows the comparison of results obtained by genotyping Guinea Bissau samples by the multilocus assay (allowing two discordant SNPs for autosomes and none for X‐chromosome) and by different combinations of PCR approaches (one or two X‐diagnostic markers in combination with PCR for loci 3L_129051 and 3R_42848). Results show that concordance with multilocus assay is <60% when using only either one or both X‐chromosome diagnostic markers, and >90% when 3 PCR approaches (either IGS + 3R + 3L or SINE + 3R + 3L) are combined. When considering the two autosomal markers separately concordance with the multilocus assay is 82%–83% for locus 3L_129051 in combination with one or both X‐diagnostic markers and 85%–87% when considering locus 3R_42848 (Table S8).

**TABLE 4:** Comparison between classification of *Anopheles coluzzii* and *An. gambiae* from Guinea Bissau based on combinations of PCR assays and on multilocus mass‐spectrometry assay. When more than one PCR assay is used, admixed individuals (adm) include all specimens for which the classification is not consistent among different markers. In the multilocus assay, adm individuals include all specimens with at least ≥1 locus on chromosome‐X and/or ≥2/14 autosomal loci not consistent with the other loci

		Multilocus genotype				Concordance
		Co	Ga	adm	Total	
IGS	Co	5	–	6	11	0.566
	Ga	–	37	40	77	
	adm	–		18	18	
SINE	Co	5	1	7	13	0.462
	Ga	–	36	49	85	
	adm	–	–	8	8	
IGS+SINE	Co	5	–	4	9	0.585
	Ga	–	36	39	75	
	adm	–	1	21	22	
IGS +3L+3R	Co	5	–	2	7	0.915
	Ga	–	33	3	36	
	adm	–	4	59	63	
SINE +3L+3R	Co	5	–	1	6	0.915
	Ga	–	32	3	35	
	adm	–	5	60	65	
IGS+SINE +3L+3R	Co	5	–	1	6	0.915
	Ga	–	32	3	35	
	adm	–	5	60	63	
Total		5	37	64	106	

Abbreviations: adm, admixed; Co, *An. coluzzii*; Ga, *An. gambiae*.

Genotyping of specimens from Burkina Faso (*N* = 146 *An. coluzzii* and 39 *An. gambiae*) led to 96.8% and 90.3% successful amplification for 3L‐ and 3R‐locus, respectively. Among successfully genotyped specimens, only three were characterized by autosomal patterns not consistent with species identification: (a) One *An. gambiae* specimen with a *An. coluzzii*‐specific 3L homozygous pattern, resulting heterozygous after sequencing, (b) one *An. coluzzii* specimen with 3L an 3R heterozygous patterns (confirmed by sequencing for locus 3L), and (c) one *An. coluzzii* specimen with 3R heterozygous pattern, for which sequencing was not successful.

Considering specimens from Guinea Bissau and Burkina Faso altogether, four discordances between PCR and Sanger sequencing were revealed (Table S7). Of these, three specimens produced a heterozygous sequence for locus 3L_129051, while showing a homozygote (AA) PCR‐pattern, either due to a preferential amplification of the allele A or, as explained above, by the presence of a polymorphism in the inner primer binding site. One specimen genotyped by sequencing as homozygote CC for locus 3R_42848 produced a heterozygote PCR‐pattern, suggesting an aspecific binding of the A‐specific interior primer.

## 4 DISCUSSION

### 4.1 Novel tools for *An. coluzzii* and *An. gambiae* genotypization/identification

In this study we developed and validated two complementary genotyping approaches to allow discrimination among *An. coluzzii*, *An. gambiae* and admixed individuals (including F1 hybrids) and to overcome the limitations of conventional chromosome‐X‐linked markers (i.e., IGS and SINE).

The first approach‐ based on the genotyping of selected 26 AIMs among the 506 identified by Miles et al. (Ag, 1000G Consortium, 2017) – allows identifying pure *An. coluzzii* and *An. gambiae* specimens and F1 hybrids, as well to evaluate levels of admixture between the two species in field populations (Figure S1), consistent with results obtained using the full set of AIMs. The assay represents a significant advancement compared to the Divergence Island SNP’ (DIS) assay developed by Lee, Marsden, Nieman, et al., (2013) for the same purpose. First, it includes a higher number of species‐specific SNPs (i.e., 26 instead of 15). Second, the AIMs were selected across the entire species range based (on a total of 283 *An. coluzzii* and 655 *An. gambiae* from 13 Ag1000G populations) instead that across a restricted geographical range in West Africa and Cameroon as in the case of Lee et al., who could not take into consideration the species‐specific diversity in Central and East Africa. Third, the 26 selected SNPs map on all five chromosome arms (and not exclusively on chromosome‐X, −2L and −3L), covering a wider genomic area (17 Mb compared to 7 Mb covered by DIS assay) largely but not exclusively mapping in centromeric “divergence islands” (Figure 2).

The Agena iPLEX mass‐spectrometry platform utilised to genotype the 26 AIMs represents a powerful and relatively affordable tool (with costs around 2€ per specimen, if the instrument is available in‐house). It allows efficient genotyping of 26 out of the 506 AIMs identified by Miles et al. (Ag, 1000G Consortium, 2017), as confirmed by >99% concordance with Illumina sequencing results – and performs well also on low quality DNA or directly on mosquito fragments, with no need of DNA extraction. Other genotyping approaches based on different technologies (e.g., TaqMan assays, Amplicon sequencing) can be exploited in the future to genotype the 26 selected SNPs and develop even cheaper/faster methods. It is relevant to stress that, in agreement with Lee et al., we allowed for some flexibility in the definition of “pure” species (i.e., for either one or two discordant autosomal loci depending on whether SNPs on chromosomal arm 2L are included). This flexibility on is indeed arbitrary and can be adjusted also according to the total number of AIMs successfully genotyped and to the presence of specific local polymorphisms, particularly when genotyping samples from regions presently not included in Ag1000G data set. It should be noted that all 2L‐SNPs are situated close to the *vgsc* gene carrying *kdr* mutations causing resistance to insecticides and thus subjected to strong selective pressure. These *kdr* alleles appear to have arisen in *An. gambiae* but were then passed through selective introgression to *An. coluzzii*, reaching high frequencies in both species (Clarkson et al., 2014). The selective sweep of these mutations along with genetic hitchhiking of the surrounding regions has led to the loss of differentiation of this centromeric region between the two species in most of their range. While in these geographical regions these SNPs are not useful as neutral markers of introgression, their genotyping may provide interesting hints on the species susceptibility to insecticides and on past introgression events.

The second genotyping approach here presented – the bi‐locus PCR assay for simultaneous genotyping of autosomal loci 3L_129051 and 3R_42848 – is allows cost‐efficient large scale screening of autosomal admixture. Notably, AIMs on chromosome‐3 can be considered to better represent the degree of apparent inter‐specific admixture than markers on chromosome‐2, which are affected by high level of chromosomal polymorphisms on chromosomal arm‐2R and by linkage with *kdr* resistant alleles on the *vgsc* gene on chromosomal arm‐2L. The bilocus PCR assay was proved to be highly efficient and specific by comparison with results obtained by Illumina sequencing and the novel multilocus assay. Furthermore, it is cheap and easy to implement (particularly with reference to the equipment requested, i.e., a simple PCR machine) and performs well for low quality DNA particularly when the two reactions are carried out separately. Applied in combination with only one of the conventional chromosome‐X based diagnostic markers, it correctly identified >90% of specimens from Guinea Bissau (where apparent admixture between *An. coluzzii* and *An. gambiae* is known to be very advanced) as pure *An. gambiae*, *An. coluzzii* or admixed, in agreement with results by multilocus genotyping. Even when genotyping of only one of the two autosomal loci is performed in combination with chromosome‐X based diagnostic markers, this percentage is still >80% (i.e., 82% and 87% for 3L and 3R locus, respectively). When genotyping samples from Burkina Faso (where adult IGS‐hybrids are <0.2% [Pombi et al., 2017]), the bilocus PCR assay confirmed low levels of admixture expected in the region, but detected 2 *An. coluzzii* and 1 *An. gambiae* with admixture in one or both of the two autosomal loci, which was confirmed by sequencing. The bilocus PCR approach appears to be robust to PCR errors due to polymorphisms in the inner primer binding sites or to aspecific binding of inner primers, which could result in deviations from Hardy‐Weinberg equilibrium, such as heterozygote excesses or null allele homozygotes. In any case, in order to exclude the occurrence of such biases it is advisable to confirm at least a proportion of admixed genomes, identified by the bilocus assay, through sequencing of surrounding PCR fragments.

### 4.2 Admixture in the high hybridization zone in the far west of *An. coluzzii* and *An. gambiae* range

The multilocus approach confirms the very high levels of admixture in the coastal area of Guinea Bissau revealed by WGS (which lead to the “hybrid form” hypothesis; Ag, 1000G Consortium, 2017; Vicente et al., 2017) and adds relevant information to the current knowledge on *An. coluzzii* and *An. gambiae* at the western extreme of their range (Figure 2). First, individuals with a “pure” *An. gambiae* chromosome‐X pattern showing high levels of admixture on the autosomes typical of coastal Guinea Bissau are also found in coastal Gambia. This suggests that processes ongoing in Guinea Bissau involve a larger than previously realised area along the west African coast, and confirms absence of a “pure” *An. gambiae* population in the coastal far‐west region. Second, 13 individuals from coastal Guinea Bissau are characterized by *An. gambiae*‐like SINE‐X200 and chromosome‐X genotypes and by heterozygous IGS (as well as admixed autosomal genotypes). This is in agreement with results from previous genotyping of X‐centromeric island in hemizygous males from coastal Guinea Bissau showing occurrence in this geographic region of recombination among rDNA repeats in otherwise pure *An. gambiae* chromosome‐X background (Caputo et al., 2016). Third, individuals with “pure” *An. coluzzii* chromosome‐X pattern and low levels of admixture on the autosomes are found in sympatry with the former ones both in coastal Guinea Bissau and in coastal Gambia (although with opposite relative frequencies), possibly suggesting (partial?) isolation or directional introgression between the two populations. Fourth, putative F1 hybrids (i.e., individuals with most selected chromosome‐X SNPs at the heterozygous state) are observed in coastal Guinea Bissau and sporadically in inland Guinea Bissau and coastal Gambia, suggesting ongoing hybridization either with a residual *An. gambiae* population or with *An. gambiae* migrated from inland areas. Fifth, populations of *An. gambiae* and *An. coluzzii* from inland Guinea Bissau are characterized by very low levels of admixture both on the X‐chromosome and on the autosomes, and populations from inland Senegal show a virtually pure AIM‐pattern, confirming preliminary evidence that the evolutionary process ongoing along the coast is very limited inland. More detailed analysis of the data set does not pertain to this study, but the above evidence clearly indicates the high value of a multilocus approach to study evolutionary processes ongoing between and within these two major malaria vectors. This is instrumental in order to address this issue not only in the “far‐west” but also in other regions in west Africa (e.g., to define the limits of the high hybridization zone), as well as in East Africa, where WGS data highlighted high levels of autosomal admixture in *An. gambiae* in the (apparent) absence of sympatry with *An. coluzzii* (Ag, 1000G Consortium, 2017).

As detailed in the previous paragraph, the exploitation of the novel bilocus PCR assay in association with conventional chromosome‐X linked species diagnostics allows a scenario to be depicted similar to the one obtained by the multilocus approach, i.e., higher apparent autosomal admixture in *An. gambiae* coastal populations from Guinea Bissau and The Gambia compared to sympatric *An. coluzzii* and low/no admixture in inland populations from Guinea Bissau and Senegal, respectively.

## 5 CONCLUSIONS

### 5.1 Results from Ag1000G and from several studies

in the last decades highlight a complex relationship between *An. coluzzii* and *An. gambiae* throughout their range, whose relevance in malaria epidemiology and in vector control has still to be fully recognised. This is largely due to lack of easy to implement and affordable tools to detect signature of genomic admixture on a large scale and to associate these to epidemiologically relevant characters (e.g., *Plasmodium* infection rate, feeding habits, host‐preferences, susceptibility to insecticides).

We suggest that knowledge on these topics can be rapidly advanced by large scale application of the biallelic PCR assay here presented in association to one of the two conventional chromosome‐X‐linked diagnostics. So far, hybridization has been only revealed by unusually high number of specimens showing an IGS heterozygous pattern (Caputo et al., 2008, 2014; Ndiath et al., 2014; Niang et al., 2014; Nwakanma et al., 2013; Oliveira et al., 2008; Vicente et al., 2017). On the other hand, the high level of autosomal admixture detected by WGS in Kenya would have never been detected by routine X‐linked diagnostics (Ag, 1000G Consortium, 2017). We envisage that applying this new genotyping approach in the frame of large scale field studies carried out for malariological purposes (e.g., to monitor susceptibility to insecticide in populations target of vector control activities of shifts in behaviours following LLIN massive implementation) will allow to detect possible signatures of admixture between the two species. Whenever genotyping results will be found not to be consistent between the autosomal and the X‐chromosome markers, further analysis could be performed using the novel multilocus mass‐spectrometry assay to investigate levels of introgression across the population and the genome and to pave the way to deeper demographic studies based on larger set of neutral markers. We expect that the application of this approach on samples from several geographic regions from where little is known about *An. coluzzii* and *An. gambiae* has a great potential to provide a continental perspective of interspecific relationships and on speciation processes. In addition, both the bi‐ and multilocus the assay allow to detect admixture in male samples hemizygous for the chromosome‐X markers, opening the unprecedented opportunity of studying the bionomics of hybrid/admixed males, with particular to swarming and mating behaviour.

More ambitiously, we propose that the two selected autosomal SNPs become complementary diagnostic markers for *An. coluzzii* and *An. gambiae*. Indeed, we are aware that this could question the actual identity of the two species, which were initially described as molecular forms (della Torre et al., 2001) and eventually raised at the species status (Coetzee et al., 2013) based on IGS‐linked species‐specific SNPs. However, we believe that after 20 years from M‐ and S‐molecular form description (now corresponding to *An. coluzzii* and *An. gambiae*), the time is mature for the recognition of the need to take into account the complex relationships between and within them. Including the genotype of the two chromosome‐3 SNPs in the species definition will avoid biases in the characterization of the vectorial system, an information that may impact vector control, if the admixed populations will turn out to differ from pure ones in relation to their capacity to transmit malaria or respond to vector control measures.

## AUTHOR CONTRIBUTIONS

Conceptualization, AdT, BC, AM, KAR.; methodology, CH, EP, VP; validation, CH, VP; formal analysis AM, CDM, GB, VP; sampling, BC, JP; data curation, GB, VP; writing—original draft preparation, AdT, BC, GB and VP; writing—review and editing, AdT, AM, BC, GB, JP, KAR, VP; supervision, AdT, AM, BC, KAR. All authors have read and agreed to the published version of the manuscript.

## Supporting information

Tables S1‐S8

Supplementary Material S1

Supplementary Material S2

## Data Availability

Information on the design of the multilocus assay presented are available in Supporting Information materials; data used for the selection of SNPs are freely available from the AG1000G project (www.malariagen.com). Original SNP data obtained for populations from Guinea Bissau and The Gambia are given in Supplementary Material S2. Sequence data for 3L and 3R genotyping are available at GenBank accession numbers MW480883‐MW480891.
