# Supplementary material for: Novel genotyping approaches to easily detect genomic admixture between the major Afrotropical malaria vector species, Anopheles coluzzii and An. gambiae
Source: Mol Ecol Resour. 2021 Apr 1;21(5):1504–16. doi: 10.1111/1755-0998.13359 (PMC8252489; doi:10.1111/1755-0998.13359)
Supplement: Supplementary file 1 — Tables S1‐S8 [file MEN-21-1504-s001.docx]

**Novel genotyping approaches to easily detect genomic admixture between the major Afrotropical malaria vector species, *Anopheles coluzzii* and *An. gambiae***

Beniamino Caputo^1§^, Verena Pichler^1§^, Giordano Bottà^1,2^, Carlo De Marco^1^, Christina Hubbart^2^, Eleonora Perugini^1^, Joao Pinto^3^, Kirk A. Rockett^2^, Alistair Miles^4,5^, Alessandra della Torre^1^

**SUPPLEMENTARY MATERIAL 1**

**Supplementary Table 1 - Ag1000G samples used to test *Anopheles gambiae/coluzzi* specific multilocus assays**

**Supplementary Table 2 - Numbers of *Anopheles coluzzii* and *An. gambiae* species-specific variants**.

**Supplementary Table 3 - Results of mass-spectrometry genotyping of 29 *Anopheles coluzzi/An. gambiae* species-specific variants.**

**Supplementary Table 4 - Allele frequencies for SNPs selected for final multilocus assay using AG1000G phase 2 dataset.**

**Supplementary Table 5 - Sample collection location details for the Senegal to Guinea Bissau transects.**

**Supplementary Table 6 - Comparison between genotyping results obtained for loci 3L_129051 and 3R_42848 using the mass-array genotyping approach or the PCR-approach.**

**Supplementary Table 7 -** **Sequencing results obtained for specimens from Burkina Faso and Guinea Bissau**

**Supplementary Table 8 - Comparison between species ID obtained for 106 specimens from Guinea Bissau using the multilocus mass-spectrometry assay and X-diagnostic markers (IGS and/or SINE) in combination with 3L or 3R - PCR.**

**Supplementary Figure 1 - Results of mass-spectrometry genotyping for individuals from Ag1000G populations.**

**Supplementary Table 1- Ag1000G samples used to test *Anopheles gambiae/coluzzi* specific multilocus assays .**

| **COUNTRY** | **Number** |
| --- | --- |
| BURKINA FASO | 62 |
| UGANDA | 96 |
| GAMBIA | 12 |
| GUINEA-BISSAU | 17 |
| KENYA | 4 |
| CAMEROON | 74 |
| ANGOLA | 10 |
| GABON | 18 |
| GUINEA | 24 |
|  |  |
| LAB COLONY | 26 |
|  |  |
| **TOTAL** | **343** |

**Supplementary Table 2 - Numbers of *An. coluzzii* and *An. gambiae* species-specific variants**, ascertained by computational analysis (337), with successful primer design (122), successfully genotyped by mass-spectrometry (104) and included in the final multilocus assay (35).

| **Chromosomal arm** | **Total species-specific variants identified** | **Total species-specific variants with successful primer design** | **Total species-specific variants successfully genotyped** | **Selected species-specific variants for MS assay** |
| --- | --- | --- | --- | --- |
| X | 86 | 43 | 40 | 11 |
| 2R | 88 | 5 | 3 | 3 |
| 2L | 51 | 37 | 33 | 8 |
| 3R | 73 | 20 | 14 | 7 |
| 3L | 39 | 17 | 14 | 6 |
| **Total** | **337** | **122** | **104** | **35** |

**Supplementary Table 3- Results of mass-spectrometry genotyping of 29 *An. coluzzi/An. gambiae* species-specific variants.** Concordance between multilocus assay and Illumina sequencing on 229 individuals. Pass-rate/locus computed for individuals for which at least 50% of loci in each chromosome were successfully genotyped. Frequency of *An. coluzzii* (CO) and *An. gambiae* (GA) alleles in *An. coluzzii* samples from Burkina Faso and Angola (N=43) and *An. gambiae* samples from Burkina Faso, Cameroun, Gabon, Guinea and Uganda (N=235). In blue 3 variants excluded from the final multilocus assay.

|  |  |  | ***An.coluzzii* (Angola, Burkina Faso; N=43)** | | ***An.gambiae* (Burkina Faso, Camerun, Gabon, Guinea, Uganda; N=235)** | |
| --- | --- | --- | --- | --- | --- | --- |
| **locus** | **concordance Illumina-Sequenom (N=229)** | **per locus pass-rate (N=337)** | **freq GA-allele** | **freq CO-allele** | **freq GA-allele** | **freq CO-allele** |
| **X_18758300** | 1 | 0.99 | 0 | 1 | 1 | 0 |
| **X_19631625** | 1 | 1 | 0 | 1 | 1 | 0 |
| **X_20014696** | 1 | 1 | 0 | 1 | 1 | 0 |
| **X_20128328** | 1 | 1 | 0 | 1 | 1 | 0 |
| **X_20129288** | 1 | 1 | 0 | 1 | 1 | 0 |
| **X_22164043** | 1 | 0.75 | 0 | 1 | 1 | 0 |
| **X_22798648** | 1 | 0.96 | 0 | 1 | 1 | 0 |
| **X_23468268** | 1 | 0.99 | 0 | 1 | 0.997 | 0.002 |
| **X_24229846** | 1 | 0.96 | 0 | 1 | 0.997 | 0.002 |
| **X_24266728** | 1 | 1 | 0 | 1 | 0.997 | 0.002 |
| **2L_209536** | 1 | 0.96 | 0.848 | 0.151 | 1 | 0 |
| **2L_927247** | 1 | 1 | 0.860 | 0.139 | 0.993 | 0.006 |
| **2L_1274353** | 0.995 | 1 | 0.872 | 0.127 | 0.993 | 0.006 |
| **2L_1418210** | 0.991 | 0.96 | 0.878 | 0.121 | 1 | 0 |
| **2L_1776348** | 1 | 1 | 0.883 | 0.116 | 0.991 | 0.008 |
| **2L_1947574** | 0.995 | 0.99 | 0.883 | 0.116 | 0.756 | 0.243 |
| **2L_2431005** | 1 | 1 | 0.872 | 0.127 | 1 | 0 |
| **2R_8368731** | 1 | 0.98 | 0 | 1 | 0.985 | 0.014 |
| **2R_17854287** | 1 | 1 | 0 | 1 | 0.991 | 0.008 |
| **2R_49438586** | 0.995 | 0.95 | 0 | 1 | 0.995 | 0.004 |
| **3L_129051** | 1 | 1 | 0 | 1 | 0.980 | 0.019 |
| **3L_193173** | 0.990 | 0.90 | 0 | 1 | 0.967 | 0.032 |
| **3L_367248** | 0.968 | 0.90 | 0.166 | 0.833 | 0.980 | 0.019 |
| **3L_380974** | 1 | 0.91 | 0 | 1 | 0.981 | 0.018 |
| **3R_34264** | 1 | 0.99 | 0.011 | 0.988 | 0.995 | 0.004 |
| **3R_38161** | 1 | 1 | 0.011 | 0.988 | 0.997 | 0.002 |
| **3R_42848** | 1 | 1 | 0 | 1 | 0.997 | 0.002 |
| **3R_50590** | 0.961 | 0.83 | 0 | 1 | 0.967 | 0.032 |
| **3R_71973** | 0.995 | 1 | 0 | 1 | 0.997 | 0.002 |

**Supplementary Table 4 - Allele frequencies for SNPs selected for final multilocus assay using AG1000G phase 2 dataset.** Locus 3L-367248 is missing since not present in the the AG1000G Phase2 biallelic dataset. REF= reference allele; ALT= alternative allele

| **chromosomal arm** | **chromsome position** | **Allele frequency *An.coluzzii* REF** | **Allele frequency *An.coluzzii* ALT** | **Allele frequency *An.gambiae* REF** | **Allele frequency *An.gambiae* ALT** |
| --- | --- | --- | --- | --- | --- |
| X | 18758300 | 1.0 | 0.0 | 0.0 | 1.0 |
| X | 19631625 | 1.0 | 0.0 | 0.002 | 0.998 |
| X | 20014696 | 1.0 | 0.0 | 0.0 | 1.0 |
| X | 20128328 | 1.0 | 0.0 | 0.0 | 1.0 |
| X | 20129288 | 1.0 | 0.0 | 0.0 | 1.0 |
| X | 22164043 | 1.0 | 0.0 | 0.0 | 1.0 |
| X | 22798648 | 0.998 | 0.002 | 0.0 | 1.0 |
| X | 23468268 | 1.0 | 0.0 | 0.0 | 1.0 |
| X | 24229846 | 1.0 | 0.0 | 0.0 | 1.0 |
| X | 24266728 | 1.0 | 0.0 | 0.0 | 1.0 |
| 2L | 209536 | 0.233 | 0.767 | 0.002 | 0.998 |
| 2L | 927247 | 0.210 | 0.790 | 0.006 | 0.994 |
| 2L | 1274353 | 0.194 | 0.806 | 0.006 | 0.994 |
| 2L | 1418210 | 0.178 | 0.822 | 0.005 | 0.995 |
| 2L | 1776348 | 0.164 | 0.836 | 0.010 | 0.990 |
| 2L | 2431005 | 0.139 | 0.860 | 0.002 | 0.998 |
| 2R | 8368731 | 0.995 | 0.005 | 0.011 | 0.989 |
| 2R | 17854287 | 1.0 | 0.0 | 0.011 | 0.989 |
| 2R | 49438586 | 0.007 | 0.993 | 0.997 | 0.003 |
| 3L | 129051 | 1.0 | 0.0 | 0.024 | 0.976 |
| 3L | 193173 | 0.0 | 1.0 | 0.976 | 0.024 |
| 3L | 380974 | 0.002 | 0.998 | 0.976 | 0.024 |
| 3R | 34264 | 0.998 | 0.002 | 0.005 | 0.995 |
| 3R | 38161 | 0.996 | 0.004 | 0.004 | 0.996 |
| 3R | 42848 | 1.0 | 0.0 | 0.004 | 0.996 |
| 3R | 71973 | 0.991 | 0.009 | 0.005 | 0.995 |

**Supplementary Table 5 - Sample collection location details** (geographical coordinates and number N of specimens) for the Senegal to Guinea Bissau transects for specimens successfully genotyped.

| **country** | **site** | **LAT WGS84** | **LONG WGS84** | **N** |
| --- | --- | --- | --- | --- |
| **Western Gambia** | 008 Kartong | 13.083333° | -16.750000° | 9 |
|  | 008 Mandina ba | 13.283333° | -16.583333° | 16 |
|  | 001 Tankular | 13.416667° | -16.033333° | 8 |
|  | 009 Hamdalai | 13.566667° | -16.016667° | 23 |
|  | 009 Sare Samba Sowe | 13.583333° | -15.900000° | 25 |
|  | 002 Ker Madi | 13.527753° | -15.624046° | 8 |
|  | 004 Wellingara Kejaw | 13.550000° | -14.916667° | 12 |
|  | 003 Touba Tafsir | 13.283333° | -14.150000° | 9 |
|  | 011 Limbanbulu Yamadou | 13.416667° | -14.116667° | 10 |
| **Eastern Senegal** | 005 Jingoreh Mafy | 13.766667° | -13.666667° | 18 |
|  | 006 Laboya | 13.300000° | -13.350000° | 10 |
|  | 007 Samecouta | 12.600000° | -12.133333° | 16 |
| **Western Guinea Bissau** | Quinhamel | 11.884222° | -15.8565° | 26 |
|  | Safim | 11.956889° | -15.649222° | 67 |
|  | Antula | 11.891222° | -15.581917° | 28 |
|  | Nhacra | 11.960306° | -15.537028° | 27 |
|  | Mansoa | 12.076972° | -15.321361° | 32 |
| **Eastern Guinea Bissau** | Mandingará | 12.171361° | -15.192167° | 42 |
|  | Gambana | 12.051944° | -14.902417° | 39 |
|  | Comuda | 12.19825° | -14.628142° | 39 |
|  | Leibala | 12.362456° | -14.222222° | 69 |
|  | **Totale** | | | 533 |

**Supplementary Table 6 - Comparison between genotyping results obtained for loci 3L_129051 and 3R_42848 using the mass-array genotyping approach or the PCR-approach.** For both loci adenine (A) is *An. coluzzii* specific and cytosine (C)= *An. gambiae* specific.

|  |  | **mass-spectrometry assay** | | |  | **Total** |
| --- | --- | --- | --- | --- | --- | --- |
|  |  | **AA** | **AC** | **CC** | **concordance** |  |
| **PCR 3L_129051** | **AA** | 32 | 2 | - | 0.972 | **34** |
|  | **AC** | - | 25 | 1 |  | **26** |
|  | **CC** | - | - | 46 |  | **46** |
| **PCR 3R_42848** | **AA** | 20 | - | - | 0.991 | **20** |
|  | **AC** | - | 31 | 1 |  | **32** |
|  | **CC** | - | - | 54 |  | **54** |

**Supplementary Table 7 -** **Sequencing results obtained for specimens from Burkina Faso and Guinea Bissau**. Species diagnostics (species-ID) obtained by using IGS- or SINE-PCR as well as results for loci 3L_129051 and 3R_42848 obtained by PCR, MassArray approach or sequencing are shown. For both loci adenine (A) is the *An. coluzzii* specific and cytosine (C) the *An. gambiae* specific allele.

**Supplementary Table 8: Comparison between species ID obtained for 106 specimens from Guinea Bissau using the multilocus mass-spectrometry assay and X-diagnostic markers (IGS and/or SINE) in combination with 3L or 3R PCR.** Species definition for mass-spectrometry assay was performed as explained in the text based on 9/9 loci on chromosome X, 12/14 for autosomal loci. Ga= *An. gambiae*, Co= *An. coluzzii*, adm= admixed

|  |  | **Multilocus genotype** | | | |  |
| --- | --- | --- | --- | --- | --- | --- |
|  |  | **Co** | **Ga** | **adm** | **Total** | **concordance** |
| **IGS +3L** | **Co** | 5 | - | 4 | 9 | 0.82 |
|  | **Ga** | - | 34 | 12 | 46 |  |
|  | **adm** | - | 3 | 48 | 51 |  |
| **IGS+3R** | **Co** | 5 | - | 2 | 7 | 0.87 |
|  | **Ga** | - | 36 | 11 | 47 |  |
|  | **adm** | - | 1 | 51 | 52 |  |
| **SINE +3L** | **Co** | 5 | - | 3 | 8 | 0.82 |
|  | **Ga** | - | 33 | 12 | 45 |  |
|  | **adm** | - | 4 | 49 | 53 |  |
| **SINE+3R** | **Co** | 5 | - | 1 | 6 | 0.85 |
|  | **Ga** | - | 35 | 13 | 48 |  |
|  | **adm** | - | 2 | 50 | 52 |  |
| **IGS+SINE +3L** | **Co** | 5 | - | 2 | 7 | 0.83 |
|  | **Ga** | - | 33 | 12 | 45 |  |
|  | **adm** | - | 4 | 50 | 54 |  |
| **IGS+SINE+3R** | **Co** | 5 | - | 1 | 6 | 0.87 |
|  | **Ga** | - | 35 | 11 | 46 |  |
|  | **adm** | - | 2 | 52 | 54 |  |

**Supplementary Figure 1 - Results of mass-spectrometry genotyping for individuals from Ag1000G populations.** Rows represent individual mosquitoes (grouped by population) and columns represent 26 *An. coluzzii/An. gambiae* species-specific variants included in mass-specrometry assay (grouped by chromosome arm). Colours represent species genotype (red= *An.coluzzii*, blu=*An.gambiae*; yellow = heterozygote) . Far left columns show the species assignment according to IGS species diagnostic PCR (Fanello et al. 2002). Lines at the lower edge show the approximate physical locations of the AIM SNPs on each chromosomal arm.
